# Enhancing the rate of genetic gain in public-sector plant breeding programs: lessons from the breeder’s equation

**DOI:** 10.1007/s00122-019-03317-0

**Published:** 2019-03-01

**Authors:** Joshua N. Cobb, Roselyne U. Juma, Partha S. Biswas, Juan D. Arbelaez, Jessica Rutkoski, Gary Atlin, Tom Hagen, Michael Quinn, Eng Hwa Ng

**Affiliations:** 1 International Rice Research Institute, Los Banos, Laguna Philippines; 2 Kenya Agricultural and Livestock Research Organization, Nairobi, Kenya; 3 Bangladesh Rice Research Institute, Gazipur, Bangladesh; 4 Bill and Melinda Gates Foundation, Seattle, WA USA; 5 CGIAR Excellence in Breeding Platform (EiB), El Batan, Mexico; 6 International Maize and Wheat Improvement Center (CIMMYT), El Batan, Mexico

## Abstract

**Key message:**

The integration of new technologies into public plant breeding programs can make a powerful step change in agricultural productivity when aligned with principles of quantitative and Mendelian genetics.

**Abstract:**

The breeder’s equation is the foundational application of quantitative genetics to crop improvement. Guided by the variables that describe response to selection, emerging breeding technologies can make a powerful step change in the effectiveness of public breeding programs. The most promising innovations for increasing the rate of genetic gain without greatly increasing program size appear to be related to reducing breeding cycle time, which is likely to require the implementation of parent selection on non-inbred progeny, rapid generation advance, and genomic selection. These are complex processes and will require breeding organizations to adopt a culture of continuous optimization and improvement. To enable this, research managers will need to consider and proactively manage the, accountability, strategy, and resource allocations of breeding teams. This must be combined with thoughtful management of elite genetic variation and a clear separation between the parental selection process and product development and advancement process. With an abundance of new technologies available, breeding teams need to evaluate carefully the impact of any new technology on selection intensity, selection accuracy, and breeding cycle length relative to its cost of deployment. Finally breeding data management systems need to be well designed to support selection decisions and novel approaches to accelerate breeding cycles need to be routinely evaluated and deployed.

## Introduction

In the face of climate change, annual reductions in arable land, and localized malnutrition (Ritchie et al. 2018), plant breeding will play an essential role in feeding 9 billion people sustainably by 2050 (Godfray et al. 2010). However, many public-sector plant breeding programs in both developed and developing countries have struggled to keep pace with technological change since the Green Revolution (Jain 2010; Pingali 2012; Baranski 2015). Relatively few reliable estimates of the rate of genetic gain delivered by public plant breeding programs that serve most farmers in the developing world have been published, but these report rates of well under 1% annually under conditions faced by most smallholder farmers (Lopes et al. 2012; Masuka et al. 2017), and do not appear to have increased in the last 30 years.

Advances in statistics (Drovandi et al. 2017), quantitative and population genetics (Hill 2010; Walsh 2014), molecular biology (Moose and Mumm 2008), genomics (Dekkers 2012; Bevan and Uauy 2013; Barabaschi et al. 2016), phenomics (Finkel 2009; Cobb et al. 2013; Lobos et al. 2017), other -omics (Carnielli et al. 2015; VanEmon 2016), and most recently machine learning and artificial intelligence (Singh et al. 2016; Chlingaryan et al. 2018) offer the potential of transforming plant breeding programs toward a data-rich, evidence-based, and team-oriented process and away from the romantic tradition of an individual breeder as an artist. However, applying these new technologies to the problem of increasing the rate of genetic gain delivered in farmers’ fields will require careful attention to their impact on the parameters of the breeder’s equation.

The reduced cost and increased throughput of data acquisition means that the primary challenge facing plant breeding teams in the 2020s will not be access to modern technology [though in some developing countries that may still be the case (Badiane 2017)], but rather developing a framework for assembling relevant technological options into an optimized product development pipeline. This manuscript is intended to provide a framework to help breeding teams make wise investments by using the genetic gain equation (Lush 1937) as a guide. Commonly known as “the breeder’s equation,” the expression is a useful starting point because it articulates the parameters breeding teams manipulate as part of the crop improvement process. The equation itself is fully described by multiplying the additive genetic variation within the population (*σ*_a_), selection intensity (*i*), and selection accuracy (*r*). Eberhart (1970) later introduced the number of years per cycle (*L*) into the denominator as a way to evaluate efficiency by expressing the response to selection as change over time.

$$\Delta G = (\sigma_{\text{a}} )(i)(r)/L$$

The equation serves as a useful mental framework for considering investment priorities because it elegantly distills theory down to parameters that a breeding program aims to manipulate. With the increased complexity of plant breeding programs, in terms of the genetic and phenotypic information acquired to make selection decisions, there are important implications for the organization, management, and incentivization of breeding teams that also need to be considered. Some aspects of breeding team management will also therefore be discussed.

### Product profiles as a variety replacement strategy

Before a breeding pipeline can be designed or optimized, it must have very clear objectives with respect to the type of product it will produce. Many breeding programs refer to this as the *product profile.* The product profile describes the trait package needed to replace a specific reference or target variety. A product profile, defined by Ragot et al. (2018), is “a set of targeted attributes that a new plant variety is expected to meet in order to be successfully released onto a market segment.” Rather than an imaginative venture to design the perfect variety, a product profile attempts to focus breeding efforts on the key traits that drive incremental value creation. While a product profile could take several forms, one simplified approach is to create a document that (1) identifies an existing reference variety already grown by a majority of farmers in a region/market, (2) evaluates what farmers, consumers, and other value chain actors like about that variety, and (3) articulates the complaints key stakeholders have about the reference variety. Opinions and preferences of stakeholders can be queried via surveys and focus group discussions, and may be distilled into the product profile, a convenient list of “must-have” traits and “value-added” traits that allow a breeder to meet market demands, add value, and deliver an incrementally improved variety quickly, rather than delaying time to market by many years in an attempt to create the ideal variety. For example, a new rain-fed rice variety for South Asia might aim to replace the dominant variety *Swarna*. The product profile might specify that the new product should be 20 days shorter in duration than *Swarna*, have resistance to predominant rice blast races, out-yield *Swarna* by 10% under favorable conditions and 20% under drought conditions, and have the same grain size, shape, amylose content, and texture. This description provides breeding teams with most of the guidance they need to design the breeding pipeline and acquire needed traits. It should be noted that for many traits (e.g., yield) the product profile does not specify an absolute target but rather an advantage, in a particular target population of environments (TPE), over the variety to be replaced. This means that that variety must be used as a check in agronomic testing, and provides some guidance on the extent of testing required to detect the targeted advantage at a particular level of acceptance probability.

Based on the product profile, breeding teams can make transparent advancement decisions when a potential new variety meets all the “must-have” criteria and possesses at least one “value-added” trait that differentiates it in the marketplace. The product profile provides breeding teams a starting point for setting investment priorities that are aligned with the perspectives and opinions of multiple value chain actors. Updating of product profiles should be done regularly and involve as many diverse stakeholders as possible, but should always focus on identifying the reference variety, its attributes that must be retained in a new variety, and the opportunities for value addition. For a more thorough review of using product profiles to enable breeding decisions, see Ragot et al. (2018).

In most private-sector breeding programs, the product profile is designed by a marketing team, with critical feedback from business units, sales agronomists, and marketing departments to help translate feedback on product performance into tangible trait targets that drive adoption and align new varieties with emerging needs expressed by the farmer, processor, or consumer. Public-sector breeding institutes have not traditionally had business development units or marketing departments and therefore have relied informal relationships with customers to achieve a good understanding of the constraints faced by farmers in the target region. Without consistent and accurate feedback indicating whether breeding objectives are drifting off track, a public-sector breeder typically takes at best an academic approach or at worst a speculative approach to determining trait targets and breeding strategy (Laske et al. 2012). In an ideally, funded organization articulating well-informed breeding objectives involves market research, close and regular interaction with key stakeholders, and an understanding of the challenges associated with breeding for various characteristics. In some cases, social science teams at institutions such as CGIAR centers have developed protocols for assigning relative values to traits in interactions with particular subsets of farmers, but this has rarely carried through to the design of formal product profiles. The participatory plant breeding (PPB) and participatory varietal selection (PVS) movements, which brought farmers into breeding trials and nurseries to make selections, were an effort to provide some farmer preference and requirement information into the selection process, but relying heavily on farmer selection is neither efficient nor particularly effective, since farmer feedback was obtained only for those traits visible at the time and place of the exercise, and do not incorporate the requirements of millers, urban consumers, and other stakeholders (Atlin et al. 2001). Well-constructed and frequently updated product profiles, designed in consultation with men and women farmers, marketers, processors, and end users, distill the requirements of all stakeholders into a blueprint for varietal development.

### Resource allocation and breeding pipeline optimization

Once breeding targets have been set and the product profile developed with the input of key stakeholders, the design and optimization of a pipeline to deliver the profile are the responsibility of the plant breeder. Because most important agronomic and quality traits are polygenically inherited, only modest and incremental improvements in these traits can be made in each breeding step. Breeding for quantitative traits is best conceived as an iterative process, in which each generation incrementally increases the frequency of favorable alleles in the gene pool under selection, thereby increasing the probability of extracting a superior cultivar. Below, we consider the steps in optimizing a breeding pipeline.

### Additive genetic variation

#### Managing and maintaining genetic variance

The first step in establishing a breeding pipeline is the selection of elite parents as founders of the program. Elite germplasm can be defined as a reproductively compatible set of genotypes disproportionately enriched for favorable alleles that improve breeding value (i.e., the mean performance of the progeny of a given parent) in a particular environment or market. Breeding values are used regularly in the context of animal breeding since the breeding product is not a sire itself, but rather its progeny. A breeding value uses pedigree or genome-wide marker data to borrow information from related lines in a phenotypic data set to estimate the additive value of an individual. While a BLUP value for phenotypic performance accounts for both the additive and nonadditive genetic values of a line, a breeding value uses the relationship matrix to determine the additive value of a line, which is the primary source of genetic variance passed on to its offspring (Henderson 1976). This is critical information for parental selection decisions and determining the relative “eliteness” of a line, and is under-utilized in public plant breeding programs. For many plant breeders, the term elite is used indiscriminately without specific terms of reference to the purpose of characterizing and managing genetic diversity in a plant breeding program. “Elite” is a status best inherited from elite parents or earned through rigorous testing of the individual per se and related lines.

Selection theory suggests that the process of generating genetic gain in source populations should be treated separately from the process of product extraction. Doing so allows a breeder to manage genetic diversity, set the desired rate of genetic gain, rapidly increase the frequency of favorable alleles in source populations, and extract products from those populations as often as needed to serve the needs of farmers or stay competitive in the marketplace. In reviewing how genomic selection unifies plant and animal breeding, Hickey et al. (2017) point out that plant and animal breeding have taken very different theoretical approaches to managing elite genetic variation. They highlight that while many plant breeders focus on the intentional introgression of valuable alleles in a single line, animal breeders have practiced a quantitative population improvement approach, wherein parents of each generation are selected on the basis of high additive breeding value, determined either by pedigree or genomic methods. As a result, plant breeders often have a tendency to conflate the genetic improvement of source populations with the process of extracting commercial products from those populations in a single step that focuses on obtaining a specific complex haplotype through the manipulation of Mendelian genetics. Achieving this complex haplotype (say, a combination of several disease resistance alleles and QTLs for abiotic stress tolerance) in high frequency in a segregating population in a single step can require very large population sizes. Also, many plant breeders use complex crossing schemes with exotic or diverse materials in an attempt to bring together disparate traits of interest. This comes at a genetic cost as the constant introduction of novel alleles, the crossing with older or exotic material with low breeding value, and the extended breeding cycles from complex backcrossing reduce the response to selection per year relative to what could be achieved through selective mating of parents with high breeding value in a closed population. As such, genetic variance for an economically important trait within clearly elite breeding material is much more valuable to a breeder than is genetic variation per se. Recurrent selection schemes focused on recycling the best genetics in a closed breeding system have been demonstrated to produce high rates of genetic gain (Breseghello et al. 2009; Shelton et al. 2015), and some of the most successful plant breeding programs in the private-sector currently manage their genetic diversity in this way (Smith et al. 2015). These programs either impose strict rules about parent choice or use a closed recurrent selection approach without overlapping generations to ensure that the parents of each new breeding cycle have higher additive breeding value than the last. This is critical to ensuring that genetic gain will be achieved in each breeding cycle.

One common concern among plant breeders when confronted with this approach is that the genetic base may be too narrow, thus permitting short-term gain from selection, but at the expense of long-term progress, arguing that while the gene pool becomes increasingly elite, it also becomes increasingly inbred. Fortunately, for quantitative traits governed by complex additive genetic architectures (Boyle et al. 2017), it has been shown that even starting from a relatively small effective population size, many decades of breeding progress can still be achieved (Guzman 1998; Bernardo et al. 2006). Inbreeding is, of course, inevitable as favorable alleles become fixed in the gene pool. However, new mutations replenish genetic variance lost to allele fixation and are an important source of genetic variance for selection over long timescales (Lind et al. 2018).

High rates of genetic gain have been reported in many experiments in a range of species using closed recurrent selection schemes. In maize, Coors (1999) reviewed over 90 recurrent selection experiments and found that gains per year from reciprocal recurrent selection exceeded those produced by commercial hybrid breeders. Recurrent selection has also been very effectively used in rice, notably by the EMBRAPA upland and lowland rice breeding programs in Brazil. Breseghello et al. (2009) reported gains of 3.6% per year over three cycles of recurrent selection in upland rice. In irrigated lowland rice, Júnior et al. (2017) reported, over three cycles, an average rate of yield gain per year of 1.98%. A recurrent selection strategy for managing elite genetic diversity not only enables evolutionary processes that increase the frequency of favorable alleles over time (Falk 2010), but also permits several clear applications of modern technologies to the breeding process. Notably, the application of whole genome sequence information allows for very clear inferences to be made about the elite breeding germplasm and permits the systematic tracking and evaluation of genetic variation over successive cycles of breeding (Beissinger et al. 2014). A key step toward modernization is to gather all available data and rigorously define a core set of high-performing elite lines. Once this is achieved, a systematic characterization of the resulting material, including the generation of high-density genotype information, will create a framework for understanding, tracking, and managing elite genetic diversity.

#### Application of high-density marker data in a recurrent selection program

While the topic is worthy of its own review, the full application of high-density genotype data to a plant breeding program would include characterization of genetic variation (Wright 1931, 1933; Wang et al. 2016; Prieur et al. 2017), development of a framework for tracking identity by descent (Vela-Avitúa et al. 2015), validating the accuracy of trait markers for MAS (Li et al. 2013; Javid et al. 2015), imputation of marker information from low-density data sets (Pryce et al. 2014; Moghaddar et al. 2017), haplotype phase inference (Hess et al. 2017), and the application of optimum contribution selection to crossing decisions (Meuwissen 1997; Wang et al. 2017). For brevity, only monitoring and managing genetic variance and validating trait markers will be touched upon here, but breeding programs that manage genetic diversity carefully and rapidly recycle parents with high breeding values are well positioned to leverage insight from these data sets to appropriately manage genetic diversity such that genetic gain is maximized while the long-term erosion of genetic variance is minimized.

Effective population size (*N*_e_), a concept introduced by Wright (1931) and further refined by Crow and Kimura (1970), is one of the most commonly used indicators in population genetics and evolutionary biology for describing the rate of inbreeding in a population and assessing genetic diversity. Plant breeders should know the effective population size of their program and actively monitor it through successive cycles of breeding to ensure the long-term viability of the breeding effort. The smaller the effective population size, the faster the population will become inbred and thus no longer respond to selection. While *N*_e_ can be estimated using both pedigree and marker data (Fernández et al. 2005), the latter is preferred (Wang 2016). Knowledge of *N*_*e*_ helps design efficient selection schemes in both animal and plant breeding (Caballero et al. 1991) and based on the effective population size breeders can redesign (if necessary) the parental combinations to maintain sufficient genetic variance among future selection candidates. One strategy for using marker data to preserve the capacity for long-term genetic gain used extensively in animal breeding but with still limited use by plant breeders is optimum contribution selection (OCS) (Meuwissen 1997; Grundy et al. 1998; Sonesson and Meuwissen 2000; Hallander and Waldmann 2009). As opposed to a static parental selection and crossing strategy which does not consider the value of differential parental contribution to the next generation, OCS seeks to balance gain from selection with preventing erosion of genetic variance by employing a dynamic parental selection strategy that seeks to optimize the contribution of each selection candidate in the next breeding cycle consistent with its predicted contribution to the rate of inbreeding in the next cycle. Sonesson and Meuwissen (2000) suggested that non-random mating among the selected parents might improve the family genetic variances in the progenies of next generations and thus affect the results of subsequent selection. OCS schemes in non-random mating populations can reduce rates of inbreeding (Caballero et al. 1996), control the levels of kinship among the progeny (Caballero et al. 1996; Fernández and Toro 1999; Toro and Pérez-Enciso 1990), increase the rate of genetic response (Caballero et al. 1996, Wang et al. 2017) and ultimately help maintain genetic diversity in the selection candidates (Wang et al. 2017) through restricting the average co-ancestry of the population. As plant breeding programs shift toward more quantitative approaches, the proactive exchange of elite material and monitoring and enhancement of genetic variation will become increasingly important.

**Fig. 1:**
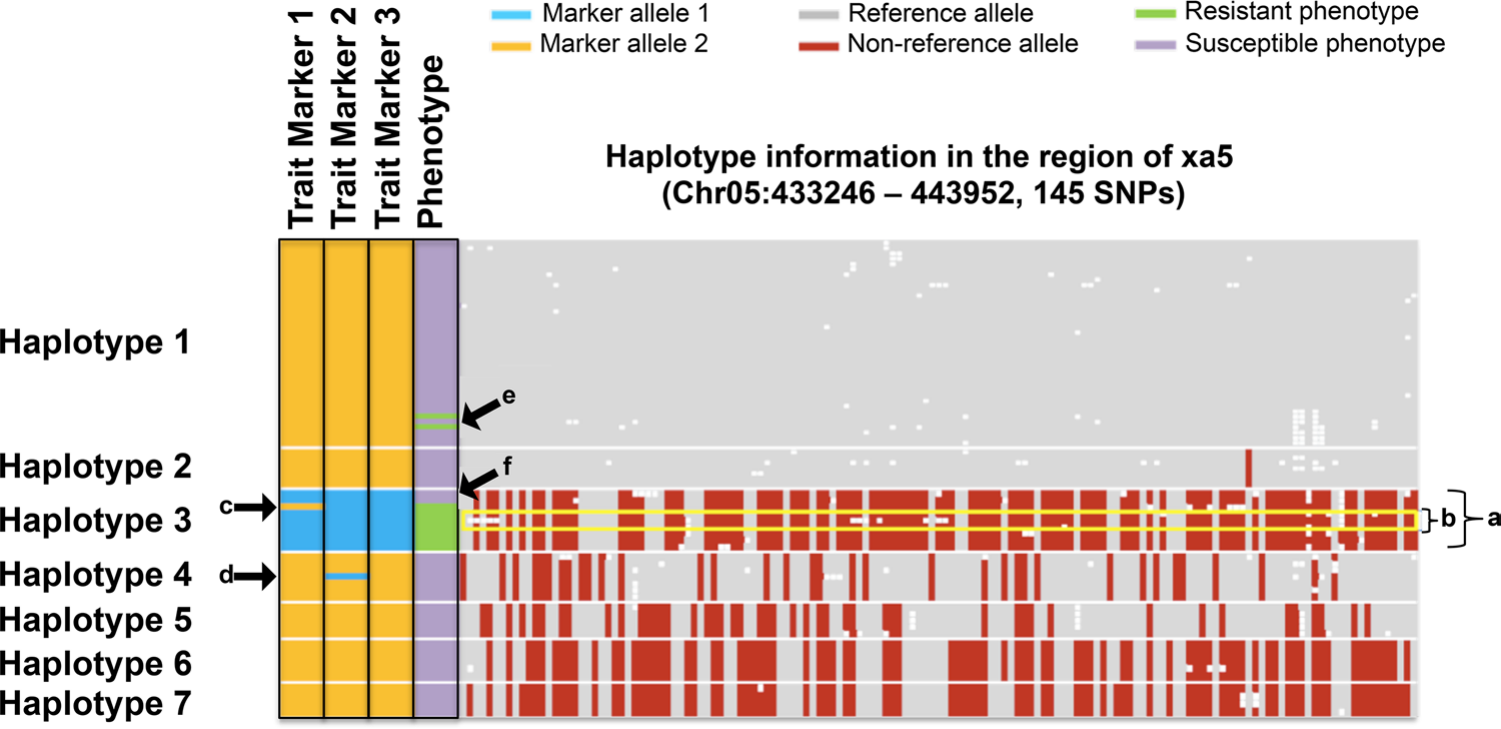
Accuracy determination of three linked SNP markers for a bacterial leaf blight resistance gene (xa5) among resequenced lines in the IRRI Irrigated breeding program. **a** The haplotype of the validated donor line indicates the known resistant haplotype. **b** IRBB 60, IRBB 61 and IRBB 64 as validated trait donors for the resistant xa5 allele. **c** A breeding line with a negative marker score for trait marker 1, but harboring the QTL[+] haplotype (i.e., a false negative for marker 1). **d** A breeding line with a positive marker score for trait marker 2, but possessing a QTL[−] haplotype (i.e., a false positive for trait marker 2). **e** Line exhibiting a favorable phenotypic response from another unlinked locus or phenotyping error. **f** Seed source variation (error) between the phenotyped source and the sequenced source showing susceptible phenotypes among the QTL[+] haplotype group

In addition to monitoring and managing the effective population size of the program, another key advantage to applying high-density marker information in a recurrent selection breeding scheme is that it provides clear information about the haplotypic diversity available within an elite gene pool, and the association of that diversity with key trait markers targeted for marker-assisted selection. Reviewed by Cobb et al. (2018) in a companion paper, the effective application of MAS to a breeding program depends on having accurate marker systems capable of properly identifying QTL[+] and QTL[−] lines. The use of diagnostic markers based on the functional polymorphism is obviously ideal (Barr 2009), but many times such knowledge is unavailable. Using linked markers then becomes necessary, but the efficacy of a linked marker depends on linkage phase, allele frequency, genomic region, and the nature of the QTL interval. With sequence data available on lines representing the key pedigrees within a breeding program, haplotypes can easily be determined and breeding program specific error rates for markers of potential value can be determined (see Fig. 1). Quality metrics for assessing the value of a marker in the context of an elite breeding program had recently suggested by Platten et al. (2019).

#### Germplasm enhancement and pre-breeding

Careful management of genetic variation, as important as it is, will not compensate for when heritable variation is simply not present among elite lines—for example when a major disease resistance allele is not present in the elite gene pool. In these cases, pre-breeding or germplasm enhancement is necessary and careful strategies need to be deployed in order to introgress the needed variation without unnecessarily contaminating the elite breeding gene pool with parents of average or low value. The need to focus this effort and to resource it independently is discussed above, but some tactical considerations for pre-breeding activities are worth noting.

The most common approach to pre-breeding is to use genetic mapping to identify QTL for key phenotypic variation and to introgress those QTL into the elite gene pool. While genetic mapping is a powerful strategy to resolve complex traits into discrete Mendelian units (quantitative trait loci or QTL; Barr 2009), it is important to recognize that the effect size of the QTL affects the deployment strategy substantially. QTLs with small effects rarely warrant marker-assisted introgression. The expense of fine mapping and marker development for small-effect QTL is rarely worthwhile when they constitute only a small proportion of the genetic variation for the trait in existing breeding germplasm (Hill et al. 2008; Boyle et al. 2017). For many species, the primary value of exotic genetic variation is the identification and deployment of rare alleles with large effects that can be introduced into elite breeding programs via a thoughtful implementation of marker-assisted selection (see Cobb et al. 2018 for a more in-depth discussion on QTL deployment).

### Selection intensity

#### New technologies for increased selection intensity

In terms of the breeder’s equation, generating and testing more selection candidates while holding the number of selected candidates constant lead to higher selection intensity (*i*) which in turn increases the rate of genetic gain. Selection intensity can also be increased by selecting fewer parents; however, it is usually more advisable to determine the number of parents to select based on whether the objective of the breeding program is long- or short-term genetic gain (Bernardo et al. 2006). Thus, increasing *i* by way of increasing population sizes requires that either budgets be increased, or a reduction in the cost of testing each selection candidate.

As budgets are usually fixed, several new technologies have been proposed to help reduce the cost of testing as a way to increase *i*, thereby increasing gain from selection. For example, genomic selection proposed by Meuwissen et al. (2001) could be used to increase the total number of selection candidates with a fixed budget if genotyping is less costly than phenotyping. Further, sparse testing designs, wherein individual lines are unreplicated or partially replicated across locations, but relatives are randomized among locations to allow estimates of haplotype x environment effects, can reduce the replication of selection candidates within and across environments. This reduces field costs and would allow a larger number of selection candidates to be tested (Endelman et al. 2014; Roorkiwal et al. 2018). Simulation studies by Lorenz (2013) and Riedelsheimer et al. (2013) found that the application of genomic prediction generally led to greater response to selection because phenotyping all selection candidates, even at reduced levels of replication, increased both the accuracy and intensity of selection.

Aside from genomic selection, low-cost screening of large population sizes prior to more advanced yield testing based on conventional MAS and/or phenotyping remains useful and can lead to high *i* for specific traits such as disease resistance. However, it is possible that selection imposed in early generations or prior to more extensive phenotyping could actually reduce gain from selection for traits evaluated in more advanced stages of testing such as grain yield and quality due to unfavorable genetic correlations between traits evaluated in early generations and traits evaluated subsequently. For example, intense selection for early flowering in early generations would actually lead to less response to selection for grain yield in subsequent stages due the effect that direct and indirect selection has on reducing the genetic variance (Bulmer 1971) prior to inter-mating. Additionally, intense selection based on traits or markers prior to advanced testing could reduce the selection candidate population from thousands to hundreds, substantially reducing *i* and gain from selection for traits tested at advanced stages. Thus, large-scale screening and selection prior to advanced testing should be done with careful consideration of the impacts it will have on the overall gain from selection for all traits of interest. It should be noted that in some breeding programs where high priority “must-have” traits are at low frequency among elite lines, it may be worth sacrificing the highest yielding individuals, in order to increase the frequency of such a trait in the short term before refocusing on agronomic performance.

In addition to marker-based solutions to increasing selection intensity, one emergent technology that could enable greater gain from selection by increasing *i* is high-throughput phenotyping (HTP). Rapid and low-cost data capture using sensors can be converted into variables that may be useful as secondary traits for indirect selection either on their own or as part of pedigree or genomic prediction schemes. Studies by Rutkoski et al. (2016) and Sun et al. (2017) found that pedigree and genomic prediction accuracies for grain yield in wheat approached those of direct phenotypic selection when data on secondary traits measured using an aerial HTP platform were utilized. This suggests that it may be possible to increase gain from selection with a fixed budget by increasing the number of selection candidates planted in yield trials, phenotyping secondary traits using HTP, and only harvesting a portion of the yield trial. Such an approach may be especially advantageous where the cost of harvesting is high, such as when trials are conducted in remote locations and/or when the harvesting must be done manually.

It should be noted, however, that genetic gain is not linearly related to the number of selection candidates. Increasing the number of selection candidates tenfold, from 10 out of 100 to 10 out of 1000, increases standardized selection differential, and therefore genetic gain, by only 52% (Table 1). The difficulty and expense of trying to increase genetic gain through increased selection intensity relative to reduced cycle time are discussed in more detail later.

**Table 1:** Relationship between proportion selected, standardized selection intensity (*i*), and genetic gain

Effective population size (*N*_e_)	Proportion selected	Total population	Standardized selection differential	Genetic gain relative to *N* = 100
10	0.1	100	1.75	1
10	0.05	200	2.063	1.18
10	0.01	1000	2.665	1.52
10	0.005	2000	2.892	1.65
10	0.001	10,000	3.367	1.92

### Selection accuracy

Phenotyping is currently (and is likely to remain) the most expensive component of a plant breeding operation. HTP may have some applications for helping to increase selection intensity, but may have more important effects on enhancing selection accuracy. The value of improvements in phenotyping is usually expressed by citing increases in broad sense heritability (*H*^2^). While this metric is useful for comparing two phenotyping strategies, it is important to recall that genetic gain is proportional to the genetic accuracy, which is the square root of the narrow-sense heritability (*h*^2^). This has big implications for deciding how to invest a breeding program’s limited resources. For most breeding programs, the best way to increase heritability is to better sample the targeted population of environments by increasing the number of yield trial locations. This turns out to be a very expensive option and is limited by physical capacity and partnerships as much as it is by budgets. Thus, most innovations in phenotyping have focused on extracting more information from existing yield trials. This can lead to making large expenditures in capital equipment and/or digital devices, and as such, careful thought needs to be put into a phenotyping strategy for a breeding program (Cobb et al. 2013).

#### HTP and the digitization of data collection

Breeding programs in recent years have witnessed an influx of engineering solutions such as robotics, imaging systems, and unmanned vehicles to increase the throughput of phenotyping (Tattaris and Reynolds 2016; Awada et al. 2018). These solutions have provided significant cost savings and efficiency gains to breeding programs via automation of routine processes, addition of new data types for more unbiased selection, and the scaling up of data collection processes at a reduced cost. While these technologies are the focus of several reviews, one unifying principle across them that is seldom discussed is the value of digitized data collection. Digitization of breeding data, both phenotypic and genotypic, is crucial for breeding programs to scale up in the twenty-first century. With cost of computing power decreasing exponentially, it has enabled genotyping data (already in digital form) to be used with ease and minimal manual curation. However, this is not the same for phenotypic data as many public plant breeding programs to date are still relying on paper and pen to record and subsequently transcribe data into usable formats (Rife and Poland 2015). Manual data collection strategies like this render phenotypic data collection expensive, labor-intensive and error-prone. Mackay and Caligari (1999) went so far as to simulate the effect of common typographical errors on the rate of genetic gain and found significant reductions in response to selection for error rates as low as 1%. Paradoxically, they showed that in many cases, response to selection would actually increase if selection intensity is reduced simply because misrepresented data points are disproportionately found among selected candidates. The idea to digitize phenotypic data collection in plant breeding was discussed as early as the 1990s by Berke and Baenziger (1992), but due to the high cost of field-ready electronic devices at the time and lack of technical skills to deploy them, adoption rates of digital field data collection remained low until the smartphone revolution of last decade. The advent of high-performance computing that fits in the palm of our hand has led many teams to develop software that permits the digital collection of phenotype data in the field. One such example of this is the PhenoApps (http://phenoapps.org/apps/). The PhenoApps tools are completely open-source Android-based applications that aid breeding programs in digitizing data collection and maintaining the chain of custody (Rife and Poland 2015). Additional software tools include Phenobook (Crescente et al. 2017) and the PhenoTyper (Köhl and Jürgen 2015).

#### Breeding data management

It should be intuitively recognized that digitizing the collection of data is actually a fairly easy problem to solve relative to the grand challenge of bringing all sources of breeding data together to inform selection decisions. Platforms need to be built that bring phenotype data, genotype data, pedigree data, and ultimately climate and weather data together in interoperable ways that permit the analysis of multi-year, multi-location, and multivariate information on generations of related lines. Sophisticated breeding informatics systems must be created and incorporated into breeding workflows to enable better predictions on the performance of different genotypes across various environments. These systems need to be robust in their architecture, computationally powerful, accessible even in remote locations, and user-friendly. There are currently very few systems available to public-sector breeding programs at the time of this publication fully capable of accomplishing this task, and no single system is in widespread use across public plant breeding programs; however, Rathore et al. (2018) provide a thorough overview of some of the strongest contenders. A fully functional and integrated system should seamlessly: (1) define, display, and permit the revision of distinct breeding zones, their target markets, and corresponding product profiles for more accurately selecting and advancing germplasm, classifying trial locations, and advancing selection candidates against defined criteria; (2) permit simplified workflows for trial design, trial management, and phenotyping procedures that allow breeding teams to quickly and easily implement robust statistical designs; (3) employ yield trial analytics with phenotypic spatial correction, location data quality evaluations, multi-location analytical capabilities, and the generation and display of pedigree estimated breeding values (pEBV) and genomic estimated breeding values (gEBV); (4) intuitively integrate pedigree, genotype, phenotype, and climate information across years, geographies, and generations to more accurately predict, summarize, and interpret variance components such as genotype, location, year, agronomic management regimes, and their interaction terms; (5) simply, intuitively, and powerfully permit creating and tracking the progression of germplasm through the breeding program; and (6) effectively track breeding operations, budgets, and activities at different timescales.

**Fig. 2:**
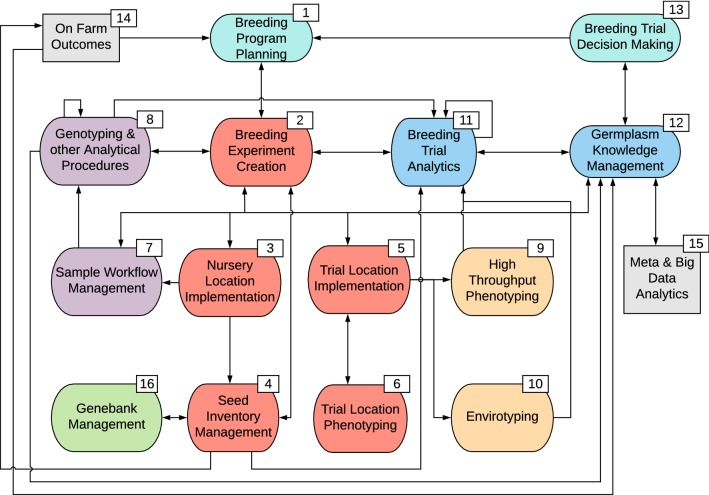
Modular design of breeding system functional capabilities. Sky blue-colored modules address breeding strategy and objectives; orange boxes correspond to the management and creation of breeding experiments; purple boxes illustrate genotyping workflows; yellow boxes for modules enabling phenotypic and environmental data collection; green boxes represent pre-breeding and gene bank management; blue boxes highlight modules for breeding analytics. Detailed explanations of numbered modules are provided in the body of the manuscript (color figure online)

Such systems would be central and formative to breeding teams endeavoring to efficiently conduct their operations, make selection decisions, optimize resources, summarize outputs, and explore the genetic consequences of breeding decisions. In order to maintain flexibility, avoid obsolescence, and leverage extensibility, these systems should ideally be modular in design with advanced data models, generic, extendable, and scalable software logic, and built using an architected approach with clear specifications. Figure 2 illustrates some suggested data and analysis modules that, together, could fully enable breeding operations.

### Cycle time

Given the complexity of the other parameters in the breeder’s equation, cycle time is the easiest to understand, cheapest to manipulate, and the most powerful parameter for increasing genetic gain. Cycle time (or generation interval) involves recycling breeding material back into the crossing block as quickly as a breeder can determine that a genotype is above average in breeding value for a desired quantitative trait. Despite its simplicity, manipulating cycle time requires careful planning and consideration of breeding strategy because a breeding team can move as quickly in the wrong direction as they can in the right one.

#### Rapid generation advance for inbred and hybrid crops

For many inbred and hybrid crops, public-sector plant breeding has maintained a strong legacy of pedigree breeding strategies focused on heavy visual selection during segregating generations. While effective for the identification of semidwarf progenies during the Green Revolution, visual selection during inbreeding is no longer effective when plant type is fixed (Atlin et al. 2017). In self-pollinated crops, approaches for rapidly developing fixed lines, such as doubled haploid (Maluszynski et al. 2003; Asif 2013) or single seed descent (Collard et al. 2017) methodologies, effectively partition genetic variation exclusively between lines, permitting the rapid evaluation and promotion of high-performing material. In general, modern breeding methods for self-pollinated crops should aim to develop fixed lines at the lowest possible cost and in the minimum possible amount of time.

Rapid generation advance (RGA) technique was first proposed by Goulden (1939) and later modified by Grafius (1965). Its most recent iteration has been in the form of “speed breeding.” (Watson et al. 2018). An RGA system shortens generation cycle through modified environments and early seed harvest in F_2_–F_6_ generations (Collard et al. 2017). These methods are regularly reviewed, and new modifications are often published (Choo et al. 1985; Snape 1989; Maluszynski et al. 2003; Forster et al. 2007; Touraev et al. 2009; Tadesse et al. 2012; Dwivedi et al. 2015; Humphreys and Knox 2015; van Ginkel and Ortiz 2018; Watson et al. 2018). Depending on the crop, the strategy, the growing season, and the budget, specialized facilities are sometimes needed to be able to accelerate generations and achieve the desired cycle time. The particular biology of the crop dictates the most successful approaches, but many techniques have been applied, including harvesting of immature seed in soybeans (Carandang et al. 2006) and pigeon pea (Saxena et al. 2017) and light manipulation in chickpea (Gaur et al. 2007), sorghum (Rizal et al. 2014) and rice (Tanaka et al. 2016). More recently, seven generations of oats and triticale had been developed in 1 year through RGA, (Liu et al. 2016) using stress to induce flowering followed by embryo rescue. Likewise Watson et al. (2018) had recently achieved six generations per year in wheat, barley, and chickpea.

**Fig. 3:**
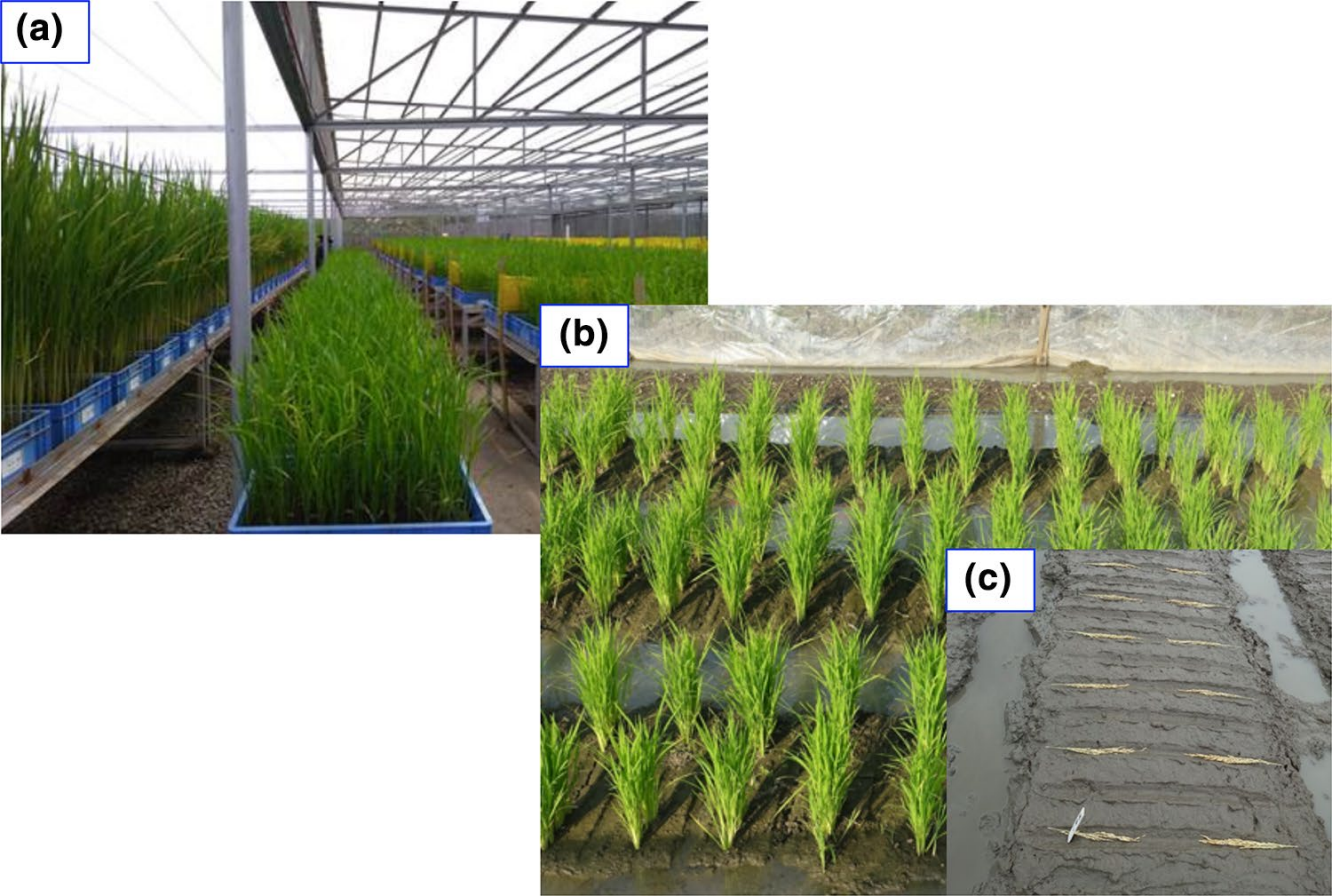
Rapid generation advance strategies at the International Rice Research Institute. **a** RGA greenhouse facility. **b** Field RGA nursery in vegetative stage. **c** Field RGA nursery at panicle seeding

In addition to the speed, one of the primary advantages of RGA compared to other methods can be the reduction in the cost per new recombinant relative to pedigree breeding, which has a very high land and labor requirement for each fixed line generated. Embryo rescue, growth chambers, and other technologies with a high cost of entry or skilled labor requirement will be most successful where high volumes and economies of scale can spread the cost among many programs. For individual breeding programs deploying RGA as a single seed descent method, usually a greenhouse facility or even a field RGA will suffice. Such a setup was developed at the International Rice Research Institute (IRRI), reducing the cost of line fixation from hundreds of dollars (under pedigree selection) to USD $0.74 using a greenhouse-based RGA and USD $0.29 using field RGA (Fig. 3; Collard et al. 2017).

#### Emphasizing cycle time over other selection parameters

**Fig. 4:**
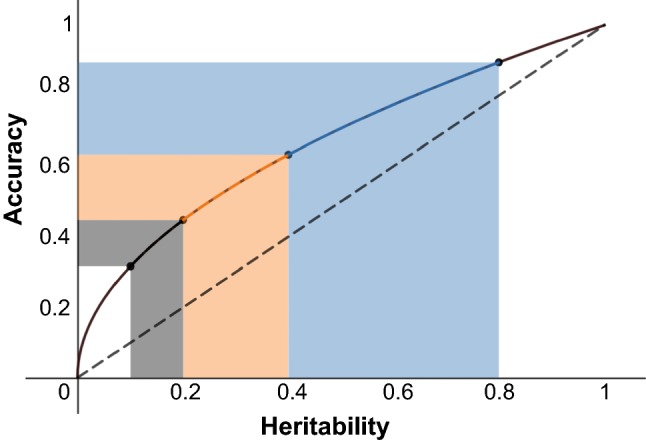
Diminishing returns on selection accuracy relative to increases in heritability. Since selection accuracy is expressed as a square root function (solid line) and not a linear function (dotted line) relative to heritability, linear increases in heritability (*x*-axis), which require significant financial investment, have diminishing impacts on selection accuracy (*y*-axis). Even modest heritabilities can command sufficient selection accuracy to drive genetic gain (orange shaded portion) (color figure online)

Accelerating breeding cycles can be the most efficient way to increase the rate of genetic gain, but it has been under-exploited in most breeding programs, which have focused more on the other three parameters of the breeder’s equation, namely heritability, selection intensity, and additive genetic variance. Unfortunately, manipulating these variables is effective initially, but they are subject to rapidly diminishing returns on investment and thus offer an expensive pathway to increased rates of genetic gain. In the case of heritability, once modest levels are achieved (in the range of 0.5–0.7) it is rarely worth the often substantial investment required to secure additional testing locations needed to increase it further. Heritability does not scale linearly with replication (i.e., doubling the number of yield trial locations will not double the heritability). Compounding this effect is the fact that neither does genetic gain scale linearly with heritability (i.e., doubling the heritability does not translate to double the response to selection). Genetic gains are proportional to the accuracy, which is the square root of heritability, and thus scales according to a square root function with heritability (Fig. 4). Thus, increasing heritability from 0.6 to 0.8 comes at significant cost but only increases the rate of genetic gain by √(0.8/0.6), or 15%. An even more extreme form of diminishing returns affects efforts to increase the rate of genetic gain by increasing population size and selection pressure. Increasing selection intensity tenfold (i.e., requiring a tenfold increase in the size of the population generated and screened if effective population size (*N*_e_) remains constant) will increase the standardized selection differential (and therefore genetic gain), by only 52%, assuming the same heritability (see Table 1). A 52% increase in genetic gain for a tenfold increase in program size and cost is an untenable solution for most breeding programs.

The impact of reducing breeding cycle length for most programs is potentially much greater than increasing heritability above about 0.6, or reducing the selected proportion below 5%. While the RGA methods described above allow breeding cycles to be reduced to three or 4 years compared to pedigree selection, there is an opportunity for breeding cycles to be shortened much further, potentially to just 1 year in the many annual cereal, oilseed, and legume crops that are not strongly photoperiod-sensitive. Most breeding programs in self-pollinated crops unnecessarily lengthen the breeding cycle by initiating selection for breeding value (i.e., selecting parents) only among highly homozygous lines. This adds significant time even relative to schemes that assess breeding value and select on non-inbred candidates. Most programs advance lines only two seasons per year and therefore would take 3 years to generate a fixed line with enough seed to enter in a yield trial before choosing to recycle lines as parents.

The breeding cycle could be greatly accelerated by selecting F_2.3_ (or S_0.1_) lines for use as parents in a rapid-cycle recurrent selection program to improve the breeding value of a population, rather than selecting highly inbred lines that can be used directly as cultivars. This amounts to a partial decoupling of parent selection for, and commercial line extraction from, elite breeding populations. This is not a novel idea; most traditional phenotypic recurrent selection programs were based on recombination of parents from the first or second generation of self-pollination, rather than highly inbred lines that can be directly commercialized. In recent years, the development and simulation of genomic selection protocols have demonstrated that breeding cycles can be completely decoupled from commercial line development, by selecting parents purely on the basis of GEBV (Heffner et al. 2009; Gaynor et al. 2016), but it has not yet been widely demonstrated experimentally that this extreme decoupling of population advancement from phenotyping will permit effective estimation of breeding value in crop breeding programs. Recurrent selection involving crossing, field testing of non-inbred progeny, and recombination in a single year is amenable to genomic selection for breeding value, especially after several cycles have been completed and linkage disequilibrium increases among parental selection candidates due to the increased relatedness of the elite source population.

An aggressive but effective single-year recurrent selection program applicable to most self-pollinated species, including rice, was developed by K.J. Frey and his students in the 1970s (Frey et al. 1988), consisting of the following steps:

1. A closed breeding population is created by inter-mating a set of selected lines chosen for high breeding value and complementary defensive and quality traits; the initial population must undergo two rounds of inter-mating to generate segregating S_0_ progeny.
2. S_0.1_ lines are generated by allowing S_0_ plants to self-pollinate.
3. S_0.1_ lines are phenotyped for yield and other traits in agronomic trials. While in some species seed may be limited at this juncture, in many of the cereal species with high tillering capacity, enough seeds for several replicates of field testing could potentially be produced from a single plant.
4. The best-performing S_0.1_ lines are inter-mated.
5. Steps 2 through 4 are then repeated for several cycles.

A genetic simulation can be used to show that radically shortening the breeding cycle from 4 years to one will greatly increase selection response even if selection intensity and heritability are reduced. Using a deterministic genetic gain simulation tool developed by Rutkoski (2018), different scenarios were simulated using the range of phenotypic values for yield from the IRRI 2016 Observational yield trial of the Drought Breeding Network. Several scenarios demonstrate the viability of ultrashort breeding cycles to drive increased genetic gain:

1. A conventional breeding program involving selection among 1000 F_5.6_ lines evaluated at *h*^2^ of 0.3, with *N* = 10 parents selected per cycle, and a breeding cycle of 4 years, over a single cycle.

This scenario represents a typical, if fairly aggressive, self-pollinated crop breeding program based on the selection of parents from among highly homozygous selection candidates.

(2) A rapid-cycle (1 year per cycle) recurrent selection program involving selection among 100 S_0.1_ lines per cycle, with *N* = 10, evaluated at *h*^2^ = 0.2, over four cycles.

This represents the fastest recurrent selection program possible in rice that still permits phenotypic evaluation each cycle. Narrow-sense heritability is assumed to be lower than the larger, conventional program posited in (1), because the quantity of seed per S_0.1_ line available for field phenotyping would likely be smaller than the quantity available from F_5.6_ lines, limiting replication over environments, and because the additive genetic variance among F_5:6_ lines is somewhat greater than that among S_0:1_ (F_2:3_) lines.

(3) A rapid-cycle (1 year per cycle) recurrent selection program involving selection among 100 S_0.1_ lines per cycle, with *N* = 10, evaluated at *h*^2^ = 0.15, over four cycles.

This scenario is identical to (2), but with an even more severe reduction in h^2^.

The results (Table 2) indicate that rapid-cycle recurrent selection in a population is only 10% as large in each cycle as in the conventional program resulted in substantially greater genetic gains, even assuming considerably reduced heritability. Even with heritability only 50% as high as in the conventional 4-year program of a single cycle of selection in fully inbred progeny, the rapid-cycle RS program, over 4 years, resulted in 50% higher rates of gain per year than the conventional program.

**Table 2:** A comparison of simulated genetic gains based on three breeding scenarios

Metrics	One cycle, 4 years/cycle, *h*^2^ = 0.3	Four cycles, 1 year/cycle, *h*^2^ = 0.2	Four cycles, 1 year/cycle, *h*^2^ = 0.15
Number of lines phenotyped per cycle	1000	100	100
Total genetic gain (kg ha^−1^)	455	954	686
Annual genetic gain (kg ha^−1^ yr^−1^)	113.75	238.5	171.5
Genetic standard deviation units per year	0.32	0.82	0.68
% genetic gain per year	2.22	4.66	3.35

A key and under-recognized element of rapid-cycle recurrent selection is that it delivers higher gains than long cycle, large population breeding at a much lower cost, because much smaller populations are evaluated each cycle and in total. In the current example, the conventional program requires the generation and phenotyping of 1000 selection candidates during the single 4-year cycle. In the rapid-cycle recurrent selection programs, 100 selection candidates are generated and phenotyped per year, for a total of 400. Perhaps 10 sublines would be extracted from each of 10 selected parents at the second and fourth cycles, to develop 100 fixed lines as candidate varieties for potential commercialization every second year. In total, the 4 years of recurrent selection would involve the creation and testing of 600 lines compared to the 1000 lines for the conventional program, while delivering much higher rates of genetic gain. In oats, Frey et al. (1988) achieved yield gains of 5.4% per cycle and therefore per year, using this protocol. Less aggressive schemes requiring 2 or 3 years per cycle have been widely used in self-pollinated crops, usually generating gains per year that are at least as high as those achieved from pedigree breeding programs. For example, Payne et al. (1986) achieved gains of 3.8% per cycle or 1.28% per year over three cycles, using a protocol requiring 3 years per cycle in oats. In soybean, Kenworthy and Brim (1979) evaluated a plan identical to that used in oats by Frey et al. (1988), but with cycles completed over 2 years. Over three cycles, an average gain of 5% per cycle or 2.5% per year was achieved for grain yield.

Shifting away from the long cycles inherent in pedigree breeding strategies toward a more accelerated 3–4-year cycle based on rapid generation advance (for inbred and hybrid crops) has the potential to make a step change in the rate at which varietal improvement is occurring. Further accelerating that by abandoning inbreeding to near fixation before selection for breeding value and redeploying the ultra-rapid single-year recurrent selection cycles will enhance the productivity of plant breeding programs by yet another step.

## Breeding program management

Successful modern breeding programs are complex and moderately expensive scientific enterprises, wherein products are developed and delivered by teams charged with delivering a steady stream of incrementally improved varieties that meet the market and farmer requirements captured in the product profile. It is a critical function of the management of breeding organizations to properly incentivize, support, and monitor the effectiveness of these teams, and to instill a culture of continuous improvement wherein the breeding pipeline is constantly assessed and new methods integrated as they become available.

### Diagramming the breeding process

**Fig. 5:**
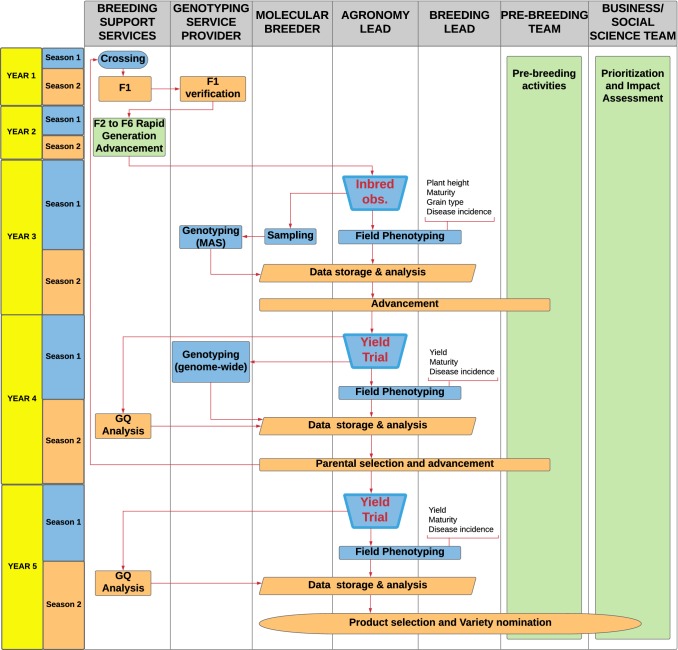
Process flow diagram for a generalized inbred breeding program based on single seed descent. Columns correspond to people, teams, or service providers. Colors indicate seasonal activities. Arrows indicate the flow of information and/or breeding material through the pipeline

One of the most valuable exercises a breeding team undertakes in initiating its continuous improvement process is to articulate and communicate the breeding strategy clearly and formally. However, this is rarely done, especially in public programs, wherein the details of the breeding strategy are often unarticulated and subject to change on the breeder’s whim. Figure 5 illustrates one example of how a multi-year RGA-based breeding strategy might be sketched out in terms of activities, accountability, and the flow of information and genetic material. While the illustrated schema is generalized and may not be applicable to any one program, it serves as a model for visualizing whom in a breeding team is responsible for which activities, and when they occur. When such a diagram is created and communicated with others, it allows breeders, pathologists, physiologists, agronomists, geneticists, and other members of the breeding team to understand where they interact with the strategy, how breeding activities fit together, identify and alleviate bottlenecks, and most importantly where innovations might be applied.

### Accountability and metrics of success

A process diagram of the breeding strategy also allows for transparency and accountability, and helps breeding management teams develop key performance indicators that assess the value of activities in delivering genetic gains. As part of a culture of continuous improvement, all breeding programs should be aiming to increase their rate of genetic gain by optimizing breeding pipelines, and demonstrating improvement by regularly measuring their outputs and progress against robust and relevant metrics. Often plant breeding programs are measured against the number of varieties nominated or released per year to the local government’s variety release pipelines. This metric often over-estimates success rates because many released varieties are never adopted by farmers (Maredia and Raitzer 2010). In private-sector breeding programs, numbers of units sold or market share are common metrics of success, but such information is difficult to obtain for public-sector programs, particularly in developing countries, and comes with the added socioeconomic complication of calculating who benefits and how equitably the benefit is distributed. Ceccarelli (2015) provides a good review of this topic and suggests that cost/benefit ratios should be used to measure plant breeding efficiency. In public-sector programs, direct estimation of genetic trend (Piepho et al. 2014), delivered under farmer management, is the likely the best method for assessing long-term breeding program effectiveness. This metric can be captured relatively easily if the breeding organization continuously evaluates newly released materials in on-farm agronomy trials.

The role of senior managers and funders in driving the efficiency of public plant breeding programs cannot be overstated. Optimization of a breeding program will not likely occur without explicit leadership and direction that incentivizes innovation, efficiency, and the delivery of genetic gains under farmer management. Moreover, it is imperative for senior management and financial stakeholders to be aware of performance against key indicators such as the average age of parental material, selection intensities (i.e., number of parents in the crossing block), selection accuracies (i.e., heritability in multi-location trials), number of lines advanced to each stage, length of the breeding cycle, and percentage of external germplasm used as parental material. Furthermore, it is worth mentioning the need for long-term, stable funding to support public breeding programs to deliver on the intended targets. The fragmented, restricted, and short-term funding models that are prevalent in most public breeding grants (especially in the developing world) incur the risk of short-circuiting breeding efficiency through frequent realignments of breeding targets or driving variable selection intensities and accuracies due to unpredictable and fluctuating budgets.

## Considerations for the future

Public-sector plant breeders and managers have a tremendous responsibility to the world as the sector often serves the communities most in need of increased rates of genetic gains. In many cases, public breeding programs are often the sole source of improved germplasm for the farmers they serve (Lopez-Pereira and Filippello 1995). Despite being immensely important, increasing rates of genetic gain in public plant breeding programs can be extremely challenging. Making changes to the breeding process in the name of increasing rates of genetic gain is often seen as very risky. Breeders often view their current pipelines as close to optimal and worry that if changes fail and the breeding program in turn fails, there will be dire consequences for the world’s most vulnerable people. This leads to a level of conservatism that can make change extraordinarily difficult. But of course change must occur in order to improve. In fact, rates of genetic gain and varietal replacement in much of the developing world have been very low since the end of the Green Revolution period, and the potential positive impact of optimizing breeding pipelines to modestly increase selection differential and accuracy, and substantially reduce breeding cycle time, greatly outweighs the risk of continuing to rely on visual selection in outmoded pedigree breeding schemes.

### Strengthening public breeding through collaboration and purposeful innovation

As plant breeding teams navigate the early twenty-first century, a few key changes need to be made to the political and social landscapes to ensure continued success. First, funding the modernization of public-sector breeding programs as a dedicated activity must take place. Given the challenges of implementing modern approaches in public breeding programs, an argument can be made for donors to fund the modernization process itself as separate and focused activity. This is the approach being taken by the Bill and Melinda Gates Foundation (BMGF) and the CGIAR through the establishment of the Excellence in Breeding Platform (EiB; https://www.cgiar.org/wp/wp-content/uploads/2018/05/SC6-04_Multi-Funder-Breeding-Initiative-update.pdf.), a clearing house and consultancy for supporting the implementation of best practices in breeding programs serving the developing world. Second, unlike private-sector organizations, public plant breeding programs struggle to leverage and benefit from the collective investment, skills and experience across crops that large transnational breeding companies can command. As a result, public plant breeding programs must form interactive communities of practice that allow them to aggregate demand and stimulate the development of low-cost genotyping, phenotyping, and open-access IT systems for storage, management, analysis, and exchange of data (Spindel and McCouch 2016). Here again, the Bill and Melinda Gates Foundation is leading an effort within the CGIAR through the High Throughput Genotyping Project (http://cegsb.icrisat.org/high-throughput-genotyping-project-htpg/) and the Genomics Open Breeding Informatics Initiative (GOBii; http://gobiiproject.org/). In an effort to deal with diminishing research funds and increased costs, many public breeding programs targeting the same agro-ecologies will need to work together in coordinated breeding networks. Through this process, and other collaborative exercises, public plant breeding communities need to come together to lift one another up to a higher standard of operating.

By coming together in the next few decades, public plant breeding communities will have an opportunity to develop a common way of understanding challenges, opportunities, and progress against standardized sets of metrics. They will need to establish industry-wide standards, common vocabulary, and shared protocols for integrating new technology into the breeding process. Breeding teams and senior management of breeding institutes can both be optimistic about opportunities to include new technologies and improved ways of operating and increasing rates of genetic gain per dollar invested. It is reasonable to expect through the thoughtful and consistent application of new technologies to breeding strategies, funders of public plant breeding efforts will gain increased confidence in the approaches being adopted and in the capacity of public plant breeding programs to meet the demands of an increasingly complex world.

## Conclusions

Public-sector plant breeding programs serving farmers in the developing world can deliver much higher rates of genetic gain if breeding programs are optimized to select for quantitative traits. Traditional pedigree breeding methods based on visual selection do not work well after plant type is fixed, and therefore, breeding pipelines serving smallholder farmers in the developing world must be modernized to optimize the key components of the breeder’s equation. Accuracy of selection for yield and other quantitative traits must be increased by testing more selection candidates in multi-location trials earlier in the breeding process, using experimental designs that effectively control field noise at low levels of within trial replication (e.g., p-rep designs). Selection intensity can be increased by replacing slow and ineffective pedigree selection, which requires visual selection of widely spaced plants in each inbreeding generation and is therefore extremely expensive in terms of time and labor, with single seed descent and bulk generation advancement techniques that rapidly move lines to fixation without selection, relying on MAS and a single visual selection step to ensure that only lines with appropriate plant type, phenology, and high-value haplotypes for disease resistance, stress tolerance, and quality are advanced to expensive multi-location trials. It should be noted, however, that returns on investment in both accuracy and selection intensity offer expensive pathways to increased genetic gain; investments in accuracy through increased replication rapidly run into diminishing returns once heritability exceeds 0.5 or so, and the relationship between genetic gain and selection intensity (i.e., population size) is roughly logarithmic rather than linear, meaning that a tenfold increase in program size is needed to double genetic gain. Modestly scaled breeding programs can achieve good genetic gains with stage one yield trials consisting of four or five well-managed p-rep trials conducted at locations representative of the TPE, with roughly 200 entries already fixed for phenology, plant type, and must-have qualitative traits, applying a selection intensity of 5% to maintain an effective population size of at least 10 per cycle.

The most underutilized pathway to increased genetic gains is likely reduced cycle time. Breeding cycles can be accelerated by immediately advancing parents selected from stage one testing for use as parents of the next cycle; additional years of testing are unlikely to increase accuracy of breeding value estimation enough to compensate for slower breeding cycles (of course, additional testing is needed before commercialization). Much greater reductions in cycle time can be achieved by selecting parents on the basis of breeding value before they are inbred to near fixation, an approach whose efficacy was confirmed in a large number of recurrent selection experiments in maize, small grains, and legume crops in the 1960s through the 1990s. Classic, closed recurrent selection breeding plans can be made much more effective by integrating genomic selection. Accelerating the breeding cycle in such schemes can deliver genetic gains equivalent to those delivered in much larger and more expensive breeding programs that cycle more slowly and apply higher selection intensity each cycle. The greatest reductions in cycle time are achievable with pure genomic selection breeding plans (e.g., Gaynor et al. 2016), in which no inbreeding or phenotyping is conducted between cycles of recombination; however, such plans require extremely large and expensively produced training populations to deliver selection accuracy, and are unlikely to be feasible in many crop species for some years.

The changes outlined in this discussion will require some initial investment on the part of most breeding programs, but will result in greater genetic gains per dollar of operating budget. More importantly than additional investment, the modernization of public breeding programs will require strong support and guidance from research managers. Research managers in the CGIAR and national breeding programs must clearly convey to breeding teams that they will be supported in and held accountable for the delivery of genetic gains in product profiles valued by farmers, processors, and consumers. External evaluation and consultancy will often be needed to help programs design and implement these changes, as programs rarely have all the necessary skills in-house.

A critical change that must be supported by research managers is shifting away from performance evaluation systems that emphasize the number of journal articles published or varieties released and toward the contribution of team members to the overall objective of generating genetic gain in the product profile. This will require understanding how individual team members contribute to the overall process and designing performance metrics accordingly.

Taken together, the improvements in product focus, selection accuracy, selection intensity, and cycle length, driven by the effective application of new genotyping, phenotyping, and decision support technologies, have the potential to raise the current rate of genetic gain in the staple food crops produced by farmers in the developing world from a current rate that is likely well under 1% annually (and in many instances not significantly different than zero) to at least 2%. In the process, farmers will be better protected against a rapidly changing climate and better able to adapt to rapidly commercializing production systems.

### Authors contribution statement

JNC and RUJ conceived and drafted the outline and ideas for the review and provided extensive editing and revision. PSB provided additional formatting and editing support. JNC, RUJ, PSB, JDA, JR, GA, TH, MQ, and EH each contributed the primary content for one or more sections. GA provided additional support for editing.
